# Prognostic Impact of COVID-19 Inflammation Score Response: A Sub-Group Analysis on Critically Ill Patients of the RuxCoFlam Trial

**DOI:** 10.3390/life15050781

**Published:** 2025-05-14

**Authors:** Manfred Weiss, Jakob Hammersen, Sebastian Rudolphi, Isabell Formann, Karl Träger, Frank G. Rücker, Beate Grüner, Andreas Allgöwer, Sebastian Birndt, Christian Fabisch, Andreas Hochhaus, Konstanze Döhner, Paul La Rosée, Frank Stegelmann

**Affiliations:** 1 Klinik für Anästhesiologie und Intensivmedizin, Universitätsklinikum Ulm, 89081 Ulm, Germany; sebastian.rudolphi@uni-ulm.de (S.R.); isabell.formann@uni-ulm.de (I.F.); karl.traeger@uni-ulm.de (K.T.); 2 Klinik für Anästhesiologie und Operative Intensivmedizin, Universitätsklinikum Augsburg, 86156 Augsburg, Germany; 3 Klinik für Innere Medizin II, Hämatologie und Internistische Onkologie, Universitätsklinikum Jena, 07743 Jena, Germany; jakob.hammersen@med.uni-jena.de (J.H.); sebastian.birndt@med.uni-jena.de (S.B.); christian.fabisch@med.uni-jena.de (C.F.); andreas.hochhaus@med.uni-jena.de (A.H.); 4 Klinik für Innere Medizin III, Hämatologie, Onkologie, Palliativmedizin, Rheumatologie und Infektionskrankheiten, Universitätsklinikum Ulm, 89081 Ulm, Germany; frank.ruecker@mutterhaus.de (F.G.R.); beate.gruener@uniklinik-ulm.de (B.G.); konstanze.doehner@uniklinik-ulm.de (K.D.); frank.stegelmann@uniklinik-ulm.de (F.S.); 5 Institute of Epidemiology and Medical Biometry, Ulm University, 89075 Ulm, Germany; andreas.allgoewer@uni-ulm.de; 6 Klinik für Innere Medizin II, Hämatologie, Onkologie, Immunologie, Infektiologie und Palliativmedizin, Schwarzwald-Baar Klinikum, 78052 Villingen-Schwenningen, Germany; paul.larosee@sbk-vs.de

**Keywords:** COVID-19, critically ill, cytokine storm, hyperinflammation, JAK inhibitors, mortality

## Abstract

This study aims to identify parameters predicting COVID-19 inflammation score (CIS) response and survival probability in critically ill patients with hyperinflammation treated with the Janus kinase (JAK) 1/2 inhibitor ruxolitinib. This is a single arm, non-randomized, open-label, phase-II study for frontline treatment in adults in the intensive care unit (ICU). Ninety-two critically ill COVID-19 patients with CIS ≥ 10 were treated in the RuxCoFlam trial (NCT04338958) with ruxolitinib between April 2020 and June 2021. Median ICU treatment duration was 15 days (range, 2–73). Out of 81 evaluable patients, 62 (77%) showed CIS reduction ≥ 25% on day 7 (CIS response). In multiple logistic regression analyses, higher CIS on day 0 (odds ratio (OR), 1.56; 95% confidence interval (CI), 1.01–2.41; *p* = 0.046) and male gender (OR, 4.76; 95% CI, 1.22–16.67; *p* = 0.024) were significantly associated with CIS response. Sixty-day survival probability was higher in CIS-responders compared to non-responders (74% vs. 32%; *p* < 0.001). Multiple Cox regression analysis revealed younger age (10-year difference) (hazard ratio (HR), 0.65; 95% CI, 0.46–0.91; *p* = 0.012) and CIS response (HR, 0.19; 95% CI, 0.08–0.45; *p* < 0.001) as significant parameters for survival probability. In conclusion, reduced risk of death in CIS-responders underlines the usefulness of CIS for the assessment of hyperinflammatory disorders, such as COVID-19, under JAK1/2 inhibitor therapy.

## 1. Introduction

Overactivation of the Janus kinase/signal transducer and activator of transcription (JAK/STAT) pathway is a common feature of proinflammatory disorders such as autoimmune diseases or viral infections. Alternatively, constitutive JAK/STAT signaling can be induced by the acquisition of gain-of-function mutations (e.g., JAK2 V617F) in hematopoietic stem cells leading to myeloproliferative neoplasm (MPN). In recent years, JAK1/2 inhibitors such as baricitinib or ruxolitinib (rux) have been approved for the treatment of rheumatoid diseases or MPN, respectively.

In COVID-19-triggered hyperinflammation, the inhibition of the JAK/STAT pathway proved as an effective therapeutic approach in several clinical trials during the pandemic [1]. Subsequently, baricitinib was approved by the U.S. Food and Drug Administration (FDA) for the treatment of COVID-19 in hospitalized adults requiring supplemental oxygen, non-invasive or invasive mechanical ventilation, or extracorporeal membrane oxygenation (ECMO). Although rux was tested in clinical studies in several stages of the COVID-19 pandemic, it is not approved for the treatment of COVID-19 triggered hyperinflammation until today [2,3,4,5,6,7,8].

The clinical efficacy and safety of rux was prospectively investigated in the RuxCoFlam trial, a single arm, non-randomized, open-label, phase-II study for the frontline treatment of adults with SARS-CoV-2-induced hyperinflammation [9]. The aim of this study was to show a mitigation of hyperinflammation in SARS-CoV-2 infected patients (pts) leading to an improved pulmonary function. In this trial, 184 patients were screened for hyperinflammation using the newly developed COVID-19 Inflammation Score (CIS). Patients with a CIS 10-16 were eligible, and clinical benefit was defined as CIS response, i.e., CIS reduction ≥ 25% on day 7 of treatment with rux, which was achieved in 71% of patients. The systemic hyperinflammation in COVID-19 is regarded to share pathophysiological similarities with virus-induced macrophage activation syndrome (MAS) or hemophagocytic lymphohistiocytosis (HLH) as potential causes for the consecutive multiorgan failure [10]. Thus, CIS is based on criteria used in HLH and defines a cohort of COVID-19 patients with hyperinflammation requiring early and specific inhibition. Moreover, it reflects changes of dynamic parameters, such as fever, ferritin, organ damage, reversal of coagulation disturbance, and cytokine suppression [10]. Diagnostic indicators for HLH are fever, splenomegaly, cytopenias, hypertriglyceridemia and/or hypofibrinogenemia, hemophagocytosis, low or absent natural killer cell activity, elevated ferritin, and soluble IL-2 receptor [11]. In COVID-19 patients, older age, comorbidities, higher disease severity status, Sequential Organ Failure Assessment (SOFA) score, white blood cell count (WBC), prothrombin time, D-dimer, ferritin, procalcitonin, and lower lymphocyte count, as well as treatments such as high-flow nasal cannula oxygen therapy, non-invasive and invasive mechanical ventilation, ECMO, and renal replacement therapy (RRT) on admission, were associated with higher mortality [12]. D-dimer, a fibrin degradation product present in blood when a blood clot is degraded by fibrinolysis, may hint at thrombosis.

In the rux multicenter trial [9], risk factors for CIS response or death have not been evaluated. Also, patients have not been differentiated in CIS-responders and CIS-non-responders regarding outcomes and effects on biomarkers. Moreover, the effects of rux in more severe, critically ill patients with a WHO scale equal to or greater than 5, i.e., patients requiring at least non-invasive ventilation or high-flow oxygen, intubation and mechanical ventilation, and/or additional organ support such as vasopressors, RRT, or ECMO, remained unclear.

Thus, to elucidate the parameters predicting CIS response and outcome in critically ill COVID-19 patients with hyperinflammation, we analyzed the sub-group of 92 RuxCoFlam patients of the rux multicenter trial [9] with a WHO scale equal or greater than 5 treated with rux on the intensive care unit (ICU) of our center.

## 2. Materials and Methods

Briefly, as in the multicenter RuxCoFlam trial (NCT04338958, first trial registration 8 April 2020) [9], we recruited patients aged ≥ 18 years hospitalized with COVID-19 pneumonia, a body temperature > 37.3 °C, and either respiratory symptoms or hypoxia SpO_2_ < 93%, as well as a study-specific CIS ≥10. Patients with uncontrolled bacterial, fungal, viral or other infection (besides SARS-CoV-2 virus), long-term use of immunosuppressive drugs, pre-existing severe organ failure and/or a survival probability of less than 6 months were excluded from study participation.

CIS reflected the sum of the following ten parameters: (i) chest X-ray or CT with hypersensitivity pneumonitis, 3 points; (ii) C-reactive protein (CRP) > 20 x upper limit of normal (ULN), 2 points; (iii) ferritin > 2 × ULN, 2 points; (iv) triglycerides > 1.5 × ULN, 1 point; (v) IL-6 > 3 × ULN, 1 point; (vi) fibrinogen > ULN, 1 point; (vii) WBC count > ULN, 1 point; (viii) lymphocytes < 1.1/μL, 2 points; (ix) body temperature > 38.5 °C, 2 points; (x) coagulation disorder: D-dimer > ULN and/or activated partial thromboplastin time (aPTT) > ULN, 1 point (adapted from [10]).

Serum concentrations of WBC, absolute lymphocyte count (ALC), CRP, ferritin, triglycerides, D-dimers, aPTT, fibrinogen, and IL-6 were routinely measured on a daily basis in the Department of Clinical Chemistry, Ulm University Medical Center. The normal range of the laboratory parameters (detection methods) were as follows: WBC 4400–11,300/μL (optical method), ALC < 1100/μL (optical method), CRP < 5.0 mg/L (turbidimetry latex assay, TURB-LAT), ferritin age-dependent 13–665 μg/L (electrochemiluminescence immunoassay, ECLIA), triglycerides < 1.7 mmol/L (ECLIA), D-dimers age-dependent < 0.64 mg/IFEU (TURB-LAT), aPTT 26–36 s (coagulometric clotting test, KOAGULO), fibrinogen 1.8–3.5 g/L (KOAGULO), and IL-6 < 7.0 pg/mL (ECLIA).

Patients were treated with rux 10 mg twice daily (BID) orally or via gastric tube in intubated patients in addition to standard of care therapy for at least 7 days with clinical and/or radiographic response assessment. In patients with unaffected (i.e., <25% decrease in CIS) or increasing CIS assessed on days 3 and 5, respectively, a 5 mg BID step-wise dose escalation was permitted at the investigator’s discretion up to a total dose of 20 mg BID. Treatment could be extended to up to 28 days if clinically indicated.

The following baseline parameters were analyzed with respect to CIS response on day 7 and survival: age, sex, body mass index (BMI), blood group, Ct-value of SARS-CoV-2, days of symptoms since disease onset, CIS on day 0, Simplified Acute Physiology Score II (SAPSII), Sequential Organ Failure Assessment (SOFA) score, and WHO ordinal severity scale for COVID-19 (Table 1) [13,14,15,16,17]. WHO ordinal scale 5 reflects patients with severe disease demanding non-invasive ventilation or high-flow oxygen, 6 intubation and mechanical ventilation, and 7 ventilation and additional organ support–pressors, RRT, ECMO [17]. Clinical symptoms, radiological results, hematology, and routine clinical chemistry were assessed locally.

### Statistical Analysis

Logistic regression and Cox proportional hazards models were used to identify prognostic variables for CIS response and survival probability, respectively. Prognostic variables were selected by using the backward elimination method. Additional covariates in multiple analysis were age (10-year difference), blood group, BMI, CIS on day 0, CIS response (>25% reduction on day 7), Ct-value of SARS-CoV-2 at baseline, sex, SAPSII, SOFA score, days of symptoms since disease onset, and WHO ordinal severity scale for COVID-19. The Wilcoxon test was used for comparing quantitative variables between time points; categorial variables were compared by means of a chi-squared test. Survival distributions were estimated using the Kaplan–Meier method, and differences between groups were analyzed using two-sided log-rank tests. An effect was considered significant if its *p*-value was less than 5%. The analyses were not adjusted for multiple testing due to their exploratory nature. Therefore, the results were interpreted with caution. All statistical analyses were performed with IBM SPSS Statistics 29.0.0.0 (IBM corporation, Armonk, NY, USA).

## 3. Results

### 3.1. Patient Disposition and Rux Treatment

**Figure 1:**
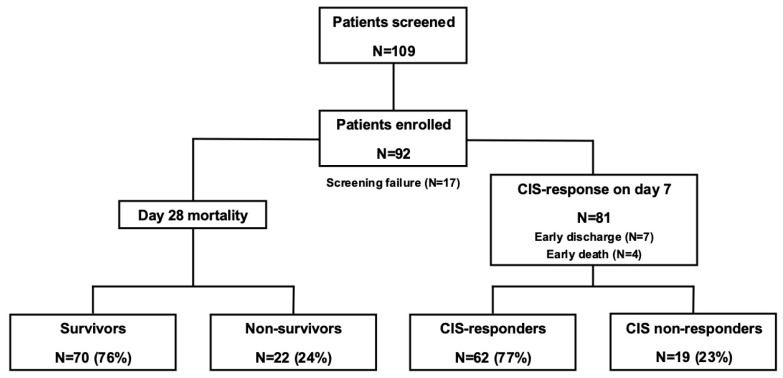
Flow chart of included patients. Trial overview. A total of 109 patients (pts) were screened, of whom 92 patients were enrolled and subsequently treated with ruxolitinib in addition to standard of care. In 11/92 patients, CIS on day 7 was not available due to early discharge (n = 7) or death before day 7 (n = 4). On day 7, 62/81 patients (77%) were CIS-responders and 19/81 (23%) were non-responders. CIS: COVID-19 inflammation score; CIS response: CIS reduction ≥ 25% on day 7 of treatment with ruxolitinib.

A total number of 109 COVID-19 patients with a CIS ≥ 10 was included in the RuxCoFlam trial at our center in the ICU of the Clinic for Anaesthesiology and Intensive Care Medicine between April 2020 and June 2021 (Figure 1). Ninety-two of 109 patients (84%) with a CIS of ≥10 (median 12) and a WHO ordinal severity scale of at least 5 were critically ill patients admitted to the ICU of the Clinic for Anaesthesiology and Intensive Care Medicine for COVID-19 treatment. Median age of the study population was 58 years (range, 25–85); 70 patients (76%) were male. A total of 16 patients (17%) required high-flow oxygenation or non-invasive ventilation, 53 patients (58%) invasive ventilation, and 23 patients (25%) ECMO. Median CIS at baseline was 12 (range, 10–16). Detailed characteristics of the 92 patients are summarized in Table 1. Median duration of ICU treatment was 15 days (range, 2–73). Rux was administered over a median of 17 days (range, 2–31). With regard to the response-guided dose escalation defined in the protocol, a maximum rux dose of 15 mg BID was reached in 26% (24/92) of the patients and 20 mg BID in 30% (28/92) of the patients until day 5. The median cumulative rux dosage was 398 mg (range, 20–1085). Standard of care treatment comprised corticosteroids in 91/92 (99%) and remdesivir in 48/92 (52%) of patients (combination of corticosteroids and remdesivir in 47/92 [51%] of patients).

### 3.2. CIS Repsonose on Day 7

**Figure 2:**
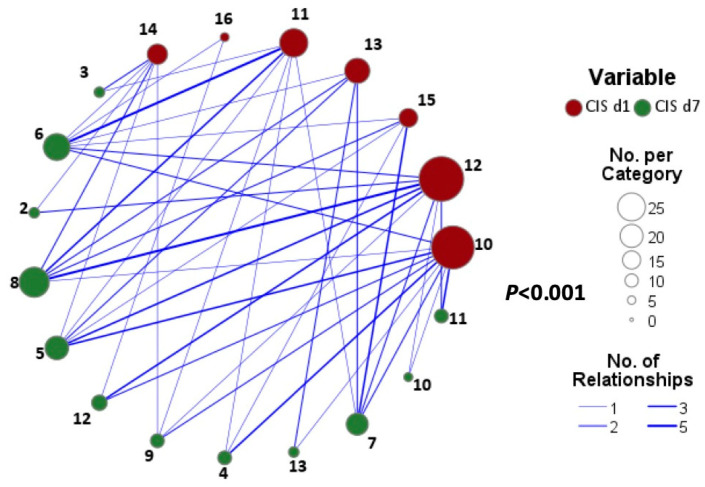
Initial dynamics of the COVID-19 Inflammation Score (CIS) between days 0 and 7. Relationship map illustrates a decrease in CIS from day 0 (red circles) to day 7 (green circles). Larger circles demonstrate a higher number of patients per CIS category, while the thickness of the blue lines represents the number of relationships between CIS on day 0 and on day 7, respectively. Median CIS on day 0 was 12 (range, 10–13) and decreased significantly to 7 (range, 3–13) on day 7 (*p* < 0.001).

At total of 81 of 92 patients (88%) were eligible for evaluation of CIS response on day 7. Median CIS on day 7 was significantly reduced compared to baseline (7 [range, 2–13] vs. 12 [range, 10–16]; *p* < 0.001) (Figure 2); 62 patients (77%) showed a CIS reduction ≥ 25% and were counted as responders, whereas 19 patients (23%) were non-responders. Of the 62 responders, 31 (50%) achieved a CIS reduction ≥ 50%. Of note, achievement of CIS response on day 7 was 91% (29/32) in patients remaining on 10 mg BID as per protocol compared to patients receiving 15 mg BID (76%; 16/21) or 20 mg BID (61%; 17/28) (*p* = 0.007).

No significant change was observed for serum levels of ferritin, triglyceride, and fibrinogen in all patients. To evaluate baseline variables predicting the achievement of CIS response on day 7, we performed simple and multiple logistic regression analyses (Table 2).

**Figure 3:**
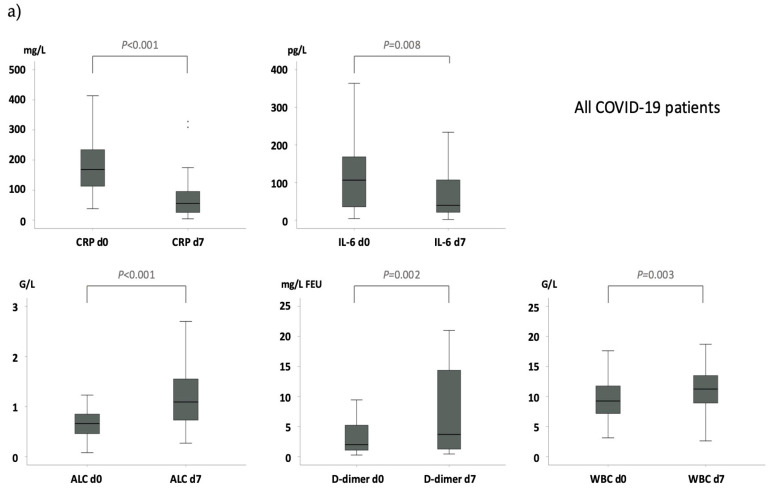

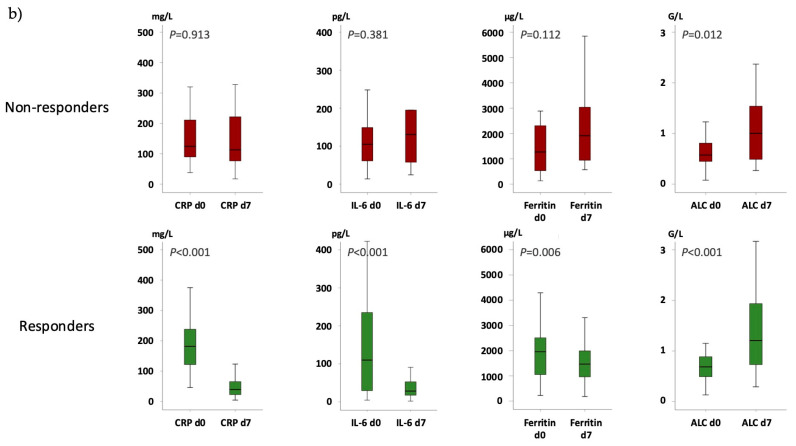
Laboratory values and COVID-19 inflammation score (CIS) response. Significant differences in the laboratory values of the CIS between baseline (day 0) and day 7 are shown by box plots. (**a**) In all patients, a significant decrease on day 7 was observed for concentrations of C-reactive protein (CRP) and interleukin-6 (IL-6), whereas a significant increase was observed for absolute lymphocyte count (ALC), D-dimer values, and white blood cell count (WBC). (**b**) In CIS-non-responders and CIS-responders, lymphocytes increased up to day 7. However, in contrast to CIS-non-responders, in CIS-responders, CRP, IL-6, and ferritin significantly decreased up to day 7.

Compared to baseline, a significant decrease on day 7 was observed for concentrations of IL-6 (median pg/mL [range]: 96 [4–1357] vs. 39 [2–4855]; *p* = 0.008) and CRP (median mg/L [range]: 157 [20–478] vs. 56 [5–328]; *p* < 0.001) (Figure 3a). In contrast, a significant increase was observed for WBC (median G/L [range]: 9.0 [3.1–19.4] vs. 11.2 [2.6–21.3]; *p* = 0.003), absolute lymphocyte count (median G/L [range]: 0.7 [0.1–3.3] vs. 1.1 [0.3–3.6]; *p* < 0.001), and D-dimer values (median mg/L FEU [range], 1.8 [0.2–21] vs. 4.1 [0.4–21]; *p* = 0.002) (Figure 3a).

Higher CIS on day 0 (odds ratio [OR], 1.56; 95% confidence interval [CI], 1.01–2.41; *p* = 0.046) and male gender (OR, 4.76; 95% CI, 1.22–16.67; *p* = 0.024) were the only significant variables predicting CIS response on day 7 in both simple and multiple models; none of the remaining baseline variables were significantly associated with CIS response on day 7. To assess the parameters driving CIS response, we compared the respective laboratory values on day 0 with those on day 7. This analysis revealed significant changes in variables positively impacting CIS in patients achieving a response (decrease in CRP [*p* < 0.001], IL-6 [*p* < 0.001], and ferritin [*p* = 0.006]; increase in absolute lymphocyte count [*p* < 0.001]). In non-responders, only an increase in absolute lymphocyte count [*p* < 0.012] was seen (Figure 3b).

### 3.3. Survival Probabilities

**Figure 4:**
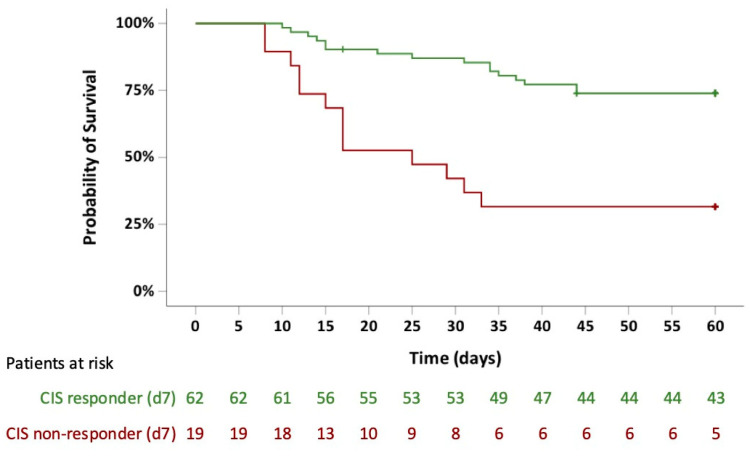
Kaplan–Meier plot for outcome according to COVID-19 inflammation score (CIS) response on day 7. Patients achieving a CIS response had an improved outcome with a 60-day survival probability of 74% vs. 32% (*p* < 0.001).

Compared to CIS-non-responders, CIS-responders had a significantly higher 60-day survival probability (32% vs. 74%; *p* < 0.001) (Figure 4). There was no difference for patients achieving CIS reduction ≥ 25% or ≥50%, respectively (60-day survival probability, 68% vs. 80%; *p* = 0.252). Accordingly, subset analyses of CRP, IL-6, ferritin, and absolute lymphocyte count on day 7 did not reveal an impact on survival probability for any of these variables.

Simple logistic regression analyses revealed a 10-year increase in age (hazard ratio [HR], 1,76; 95% CI, 1.29–2.40; *p* < 0.001), CIS non-response (HR, 4.17; 95% CI, 2.00–9.09; *p* < 0.001), and higher SAPSII (HR, 1.06; 95% CI, 1.03–1.10; *p* < 0.001) as significant variables associated with inferior 60-day survival (Table 2).

Of note, younger age (10-year difference) and CIS response were significantly favorable in multiple Cox regression analysis for day-60 survival probability (HR for age, 0.65; 95% CI, 0.46–0.91; *p* = 0.012; HR for CIS response, 0.19; 95% CI, 0.08–0.45; *p* < 0.001), outweighing established risk scores such as SAPSII, SOFA score, and WHO ordinal severity scale.

**Figure 5:**
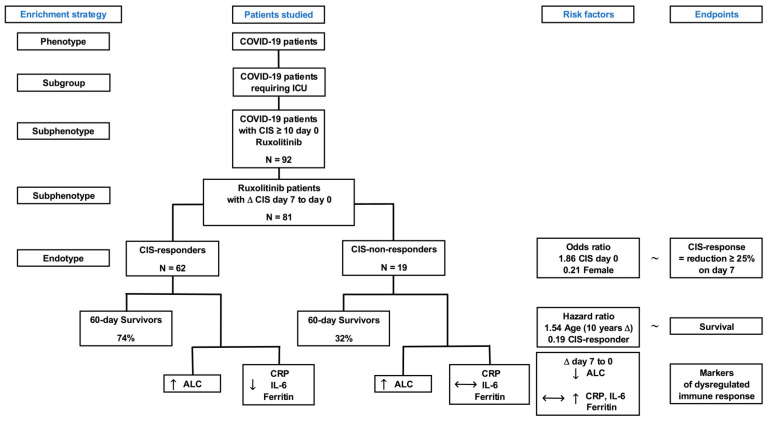
Enrichment strategy, patients studied, risk factors, and studied endpoints. Endotypes of patients are reflected by CIS-responders and CIS-non-responders. Risk factor-based effects on endpoints are presented. The expected beneficial effects of ruxolitinib (rux) on the dysregulated immune response were reduction in inflammation and increase in lymphocytes within one week. The expected and observed effects on biomarkers and outcomes underline the necessity to differentiate between CIS-responders and CIS-non-responders with respect to rux application in infection-triggered hyperinflammatory states, such as COVID-19. ALC: absolute lymphocyte count; CIS: COVID-19 Inflammation Score; ICU: intensive care unit; IL-6: interleukin 6; ~: separator between dependent and independent variables; ↑ and ↓ display a significant increase or decrease, with *p* < 0.05; 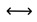 indicates no difference in the delta (Δ) from day 7 to day 0.

The results are summarized in Figure 5.

## 4. Discussion

Rux proved to be safe and effective in 184 patients with COVID-19-triggered and clearly defined hyperinflammation treated within the multicenter RuxCoFlam phase-II trial [9]. In our single-center sub-group analysis, we focused on rux response and outcome in half of the RuxCoFlam patients (n = 92) admitted to the ICU of our Clinic for Anaesthesiology and Intensive Care Medicine due to impaired pulmonary function to assess the prognostic value of CIS. More severe SARS-CoV-2 infection in these patients compared to the total RuxCoFlam study population is reflected by an increased WHO ordinal severity scale of 6 (range, 5–7) vs. 5 (range, 3–6). The key findings of our multiple analyses in critically ill COVID-19 patients were that (i) higher baseline inflammation (measured by CIS on day 0) and male gender were associated with CIS response on day 7; (ii) CIS-responders showed a superior 60-day survival probability compared to non-responders; and iii) CIS response and younger age (10-year difference) were the only two variables predicting improved outcome, with a mortality risk decreased by 81% and 35%, respectively (Figure 5).

In recent years, two randomized, double-blind phase-III trials investigating an add-on of rux to the standard of care in COVID-19 failed to reach their primary endpoints compared to placebo: First, the RUXCOVID trial analyzed the potential benefit of rux in hospitalized patients with non-severe COVID-19 [2]. In this study, low-dose rux (5 mg BID) administered for at least 14 days showed no superiority over placebo with regard to the composite primary endpoint death, respiratory failure (invasive ventilation), or need for ICU care by day 29. Second, rux given at higher daily dosages (10–15 mg BID) in the RUXCOVID-DEVENT trial of advanced COVID-19 with mechanical ventilation proved no better, in terms of efficacy, than standard of care since a reduction in the 28-day mortality rate was not achieved [8]. Consequently, rux has so far not been approved for COVID-19 treatment.

The negative results of these phase-III trials are likely due to the lack of a strategy for selecting suitable patients for treatment with rux. In contrast, the use of glucocorticoids in selected patients during the course of the COVID-19 pandemic has been successfully implemented. In the RECOVERY trial, dexamethasone decreased the mortality of hospitalized COVID-19 patients receiving oxygen alone or requiring invasive mechanical ventilation but not of those without respiratory support [18]. Thus, dexamethasone became standard of care only in COVID-19 patients needing supplemental oxygen [19]. Similar to the selection process for glucocorticoids, CIS was established for a precise evaluation of systemic COVID-19 inflammation in order to identify patients in need of rux treatment. In a pilot study of 14 patients with a CIS of at least 10 before rux treatment, 11 (79%) showed a CIS response defined as a reduction of ≥25% on day 7 [10]. The subsequent RuxCoFlam trial included COVID-19 patients with the same CIS cut-off (≥10) and yielded a similar response rate (71%), which was accompanied by a significant reduction of inflammation markers such as IFN-γ, IL-6, and IL-10 (*p* < 0.001 each) [9]. With 77%, the CIS response in our sub-group analysis of critically ill COVID-19 patients was comparable. Furthermore, we observed a decrease in the inflammation parameters CRP and IL-6 on day 7. From our point of view, these data indicate that CIS stratification is helpful in pre-selecting COVID-19 patients with hyperinflammation suitable for rux treatment.

With regard to outcome, about one half of the hospitalized patients died during the first weeks of the COVID-19 outbreak in March 2020. In a recent meta-analysis of 157 studies evaluating 948,309 patients, the case fatality rate for ICU mortality was 37% (95% CI: 35–40%), ranging up to 66% (95% CI: 60–73%) in the case of renal replacement therapy [20]. While 19% of the RuxCoFlam trial patients died until day-29 follow-up, the overall mortality rate in the ICU was 33% in our sub-group analysis. However, owing to the lack of a control group for the trial, the question of whether the addition of rux to standard of care reduced the CIS and prolonged survival remains unanswered.

Under the scope of personalized medicine, we strive for treatment of endotypes, i. e., sub-phenotypes of patients with distinct functional or pathobiological mechanisms, which preferably respond differently to a targeted therapy [21]. Regarding the heterogeneity of the phenotype COVID-19, first, a sub-group of patients was defined by severity of disease, i.e., those requiring treatment in the ICU in the present study (Figure 5); second, within this ICU sub-group, sub-phenotypes could be discriminated based on a data-driven multidimensional assessment of traits—here, by the hyperinflammatory sub-phenotype with a CIS ≥ 10. Thus, within the hyperinflammatory sub-phenotype in the present study, endotypes, preferably responding differently to rux, could be detected.

Subsequently, the effects of immunomodulatory treatments could be evaluated in terms of initial risk factors on different clinical endpoints, such as markers of immune dysregulation or mortality. This enrichment strategy may be transferred to other immunomodulatory interventions in infection-triggered dysregulations of the immune system to determine the endotypes with beneficial effects.

## 5. Conclusions

Of note, the 60-day survival probability in our study was significantly higher in CIS-responders (74%) than in non-responders (32%). This finding proves a novelty since the published RuxCoFlam trial did not include outcome data depending on response. In addition, we showed that survival probability was not further improved in patients with CIS reduction ≥50% (instead of ≥25%), confirming the ≥25% cut-off. Since subset analyses of single CIS-parameters, such as CRP or IL-6 reduction, did not translate into survival benefit alone, whole CIS parameters should be analyzed to predict the outcome under rux in future studies. Our key finding that CIS-responders and younger patients had an improved 60-day survival probability represents the second novelty of our study. This result is of great importance because in our setting of critically ill COVID-19 patients, the application of the 10-item CIS response on day 7 even outperformed established prognostic ICU scores, such as SAPSII, consisting of 15 clinical parameters to estimate the in-hospital mortality. This underlines the usefulness of the CIS for the repetitive assessment of COVID-19 hyperinflammation under rux. The performed concept of initial risk factor-based evaluation of different endpoints may be used in immunomodulatory interventions in critically ill patients to detect endotypes of patients. In conclusion, the CIS response on day 7 may serve as an ideal early surrogate marker for outcome, similar as has been shown for the ferritin–platelet ratio in a recent study on hemophagocytic lymphohistiocytosis [11] to evaluate the response after induction therapy.

## Figures and Tables

**Table 1:** Main patient characteristics including treatments and complications.

Patient Characteristics	Cases *n* (%)	Mean (SD)	Median (Min–Max)
Total	92 (100)		
Age (years)	92	58.3 (±12.2)	58 (25–85)
Gender	92		
Female	22 (23.9)		
Male	70 (76.1)		
BMI (kg/m^2^)	89	33.2 (±14.0)	29 (20–52)
Blood group	92		
O	25 (27.8)		
B	11 (12.2)		
AB	8 (8.9)		
A	46 (51.1)		
n. a.	2 (2.2)		
SARS-CoV-2	92		
ct-value	90	27.0 (±6.3)	27 (15–40)
Days of symptoms	85	9.9 (±5.2)	10 (1–24)
CIS on day 0	92	12.0 (±1.7)	12 (10–16)
Severity of disease	92		
SAPSII	92	34.4 (±9.0)	32 (15–63)
SOFA	91	5.4 (±2.2)	5 (2–12)
WHO scale	92	6.1 (±1.0)	6 (5–7)
Comorbidities *	92		
Arterial hypertension	49 (53.3)		
Cardiovascular disease	32 (34.8)		
Diabetes mellitus	23 (25.0)		
Hyperlipoproteinemia	13 (14.1)		
COPD/Asthma bronchiale	18 (19.6)		
Metabolic syndrome	9 (9.8)		
Arthritis	4 (4.3)		
Treatments *	92		
Corticosteroids	91 (98.9)		
Remdesivir	48 (52.2)		
Corticosteroids and remdesivir	47 (51.1)		
Catecholamines	62 (67.4)		
Renal replacement	8 (8.7)		
ECMO	23 (25.0)		
Maximum ruxolitinib dose	92		
10 mg	40 (43.5)		
15 mg	24 (26.1)		
20 mg	28 (30.4)		
ICU length of stay (days)	92	18.7 (±14.2)	15 (2–73)
Complications *	92		
Bacterial superinfection	65 (70.7)		
Bacteremia	41 (44.6)		
Viral superinfection/reactivation	6 (6.5)		
Fungal superinfection	33 (35.9)		
Fungemia	3 (3.3)		
Thrombosis/PAE	13 (14.1)		
Bleedings	30 (32.6)		

* Multiple answers possible. Abbreviations: BMI: body mass index; CIS: COVID-19 Inflammation Score; COPD: chronic obstructive pulmonary disease; ct: cycle threshold; ECMO: extracorporeal membrane oxygenation; n: number; n. a.: not applicable; PAE: pulmonary artery embolism; SAPSII: Simplified Acute Physiology Score; SARS-CoV-2: severe acute respiratory syndrome coronavirus type 2; SD: standard deviation; SOFA: Sequential Organ Failure Assessment; WHO scale: World Health Organization ordinal severity scale for COVID-19.

**Table 2:** Simple (**A**,**C**) and multiple (**B**,**D**) logistic regression analyses regarding CIS response on day 7 (**A**,**B**) and 60-day survival probability (**C**,**D**) under ruxolitinib treatment.

(A) CIS Response ~	Variable	Reference Group	Odds Ratio	95% CI	*p*-Value
Age (10 y difference)	continuous		0.88	[0.56–1.38]	0.567
Blood group A	categorial	All other	1.73	[0.61–4.91]	0.302
BMI (kg/m^2^)	continuous		0.99	[0.96–1.02]	0.415
**CIS d0**	**continuous**		**1.86**	**[1.21–2.84]**	**0.005**
ct-value	continuous		1.04	[0.96–1.14]	0.346
**Gender female**	**categorial**	**male**	**0.21**	**[0.07–0.66]**	**0.007**
SAPSII	continuous		0.99	[0.94–1.06]	0.814
SOFA	continuous		0.91	[0.72–1.15]	0.435
Days symptom onset	continuous		0.96	[0.86–1.06]	0.387
WHO scale 5	categorial	6 and 7	1.01	[0.35–2.93]	0.984
**(B) CIS Response ~**	**Variable**	**Reference** **Group**	**Odds Ratio**	**95% CI**	***p*-Value**
Age (10 y difference)	continuous		0.66	[0.38–1.14]	0.134
Blood group A	categorial	All other	2.65	[0.70–10.02]	0.151
BMI (kg/m^2^)	continuous		0.98	[0.92–1.05]	0.524
**CIS d0**	**continuous**		**1.56**	**[1.01–2.41]**	**0.046**
ct-value	continuous		1.03	[0.93–1.15]	0.576
**Gender female**	**categorial**	**male**	**0.21**	**[0.06–0.82]**	**0.024**
SAPSII	continuous		0.99	[0.90–1.10]	0.886
SOFA	continuous		0.83	[0.58–1.20]	0.315
Days symptom onset	continuous		1.00	[0.86–1.16]	0.975
WHO scale 5	categorial	6 and 7	0.56	[0.12–2.67]	0.467
**(C) Survival (d60) ~**	**Variable**	**Reference** **Group**	**Hazard Ratio**	**95% CI**	***p*-Value**
**Age (10 y difference)**	**continuous**		**1.76**	**[1.29–2.40]**	**<0.001**
Blood group A	categorial	All other	0.83	[0.42–1.64]	0.593
BMI (kg/m^2^)	continuous		0.99	[0.96–1.02]	0.502
CIS d0	continuous		0.95	[0.77–1.17]	0.613
**CIS response**	**categorial**		**0.24**	**[0.11–0.50]**	**<0.001**
ct-value	continuous		1.01	[0.96–1.07]	0.631
Gender female	categorial	male	1.50	[0.70–3.23]	0.301
**SAPSII**	**continuous**		**1.06**	**[1.03–1.10]**	**<0.001**
SOFA	continuous		1.07	[0.92–1.23]	0.394
Days symptom onset	continuous		1.02	[0.95–1.09]	0.561
WHO scale 5	categorial	6 and 7	0.81	[0.41–1.64]	0.562
**(D) Survival (d60) ~**	**Variable**	**Reference** **Group**	**Hazard Ratio**	**95% CI**	***p*-Value**
**Age (10 y difference)**	**continuous**		**1.54**	**[1.10–2.17]**	**0.012**
Blood group A	categorial	All other	0.80	[0.34–1.88]	0.605
BMI (kg/m^2^)	continuous		0.99	[0.95–1.02]	0.439
CIS d0	continuous		0.96	[0.72–1.26]	0.752
**CIS response**	**categorial**		**0.19**	**[0.08–0.45]**	**<0.001**
ct-value	continuous		1.06	[1.00–1.12]	0.071
Gender female	categorial	male	0.80	[0.27–2.37]	0.684
SAPSII	continuous		1.03	[0.98–1.09]	0.280
SOFA	continuous		1.01	[0.79–1.29]	0.936
Days symptom onset	continuous		1.03	[0.94–1.12]	0.577
WHO scale 5	categorial	6 and 7	0.68	[0.29–1.58]	0.370

Abbreviations: BMI: body mass index; CI: confidence interval; CIS d0: COVID-19 inflammation score on day 0; CIS response: decrease in CIS ≥ 25% on day 7 under ruxolitinib; ct: cycle threshold; Days symptom onset: Days since symptom onset; SAPSII: Simplified Acute Physiology Score II; SOFA: Sequential Organ Failure Assessment; WHO scale: World Health Organization ordinal severity scale for COVID-19; ~: separator between dependent and independent variables. In Tables C and D, Hazard ratios, 95%-CI, and *p*-values for non-significant variables are given for the last step included in the model. Variables with *p* < 0.05 are presented in bold.

## Data Availability

The datasets used and/or analyzed during this study are available from the corresponding author on reasonable request.

## Abbreviations

The following abbreviations are used in this manuscript:

aPTT	Activated partial thromboplastin time
BID	Twice daily
BMI	Body mass index
°C	Degree Celsius
CI	Confidence interval
CIS	COVID-19 inflammation score
CoV-2	Coronavirus type 2
COVID-19	Coronavirus disease 2019
CRP	C-reactive protein
Ct	Cycle threshold
CT	Computed tomography
ECMO	Extracorporeal membrane oxygenation
FDA	Food and Drug Administration
G/L	Giga/liter
HR	Hazard ratio
ICU	Intensive care unit
IFN-γ	Interferon-γ,
IL-6	Interleukin-6
JAK	Janus kinase
mg/L	Milligram/liter
MPN	Myeloproliferative neoplasm
OR	Odds ratio
Pts	Patients
RRT	Renal replacement therapy
Rux	Ruxolitinib
SAPS II	Simplified Acute Physiology Score II
SOFA	Sequential Organ Failure Assessment
SpO_2_	Peripheral arterial oxygen saturation
ULN	Upper limit of normal
US	United states
WBC	White blood cell
WHO	World Health Organization
